# Characterization of COVID-19-Related Lung Involvement in Patients Undergoing Magnetic Resonance T1 and T2 Mapping Imaging: A Pilot Study

**DOI:** 10.3390/jimaging8120314

**Published:** 2022-11-23

**Authors:** Giovanni Camastra, Luca Arcari, Federica Ciolina, Massimiliano Danti, Gerardo Ansalone, Luca Cacciotti, Stefano Sbarbati

**Affiliations:** 1 Covid-Cardiology Unit, Madre Giuseppina Vannini Hospital, 00177 Rome, Italy; 2 Radiology Unit, Madre Giuseppina Vannini Hospital, 00177 Rome, Italy

**Keywords:** COVID-19, pneumonia, magnetic resonance imaging, T1 mapping, T2 mapping, edema, fibrosis

## Abstract

Tissue characterization by mapping techniques is a recent magnetic resonance imaging (MRI) tool that could aid the tissue characterization of lung parenchyma in coronavirus disease-2019 (COVID-19). The aim of the present study was to compare lung MRI findings, including T1 and T2 mapping, in a group of *n* = 11 patients with COVID-19 pneumonia who underwent a scheduled cardiac MRI, and a cohort of healthy controls. MRI scout images were used to identify affected and remote lung regions within the patients’ cohort and appropriate regions of interest (ROIs) were drawn accordingly. Both lung native T1 and T2 values were significantly higher in the affected areas of patients with COVID-19 as compared to the controls (1375 ms vs. 1201 ms, *p* = 0.016 and 70 ms vs. 30 ms, *p* < 0.001, respectively), whereas no significant differences were detected between the remote lung parenchyma of the COVID-19 patients and the controls (both *p* > 0.05). When a larger ROI was identified, comprising the whole lung parenchyma within the image irrespective of the affected and remote areas, the COVID-19 patients still retained higher native T1 (1278 ms vs. 1149 ms, *p* = 0.003) and T2 values (38 ms vs. 34 ms, *p* = 0.04). According to the receiver operator characteristics curves, the T2 value of the affected region retained the higher accuracy for the differentiation of the COVID-19 patients against the controls (area under the curve 0.934, 95% confidence interval 0.826–0.999). These findings, possibly driven by the ability of MRI tissue mapping to detect ongoing inflammation in the lungs of patients with COVID-19, suggest that T1 and T2 mapping of the lung is a feasible approach in this clinical scenario.

## 1. Introduction

Coronavirus disease 2019 (COVID-19) is a respiratory tract infection characterized by a not uncommon development of interstitial pneumonia which can lead to severe respiratory failure and death [1]. Chest computed tomography (CT) provides accurate diagnostic and prognostic information, being able to detect and quantify COVID-19-related lung involvement [2]. Though limited by lower spatial resolution and lengthy examination time, magnetic resonance imaging (MRI) could potentially offer advantages in providing the tissue characterization of lung parenchyma [3,4]. We previously reported how a comprehensive MRI protocol including T1 and T2 mapping was able to detect differences between affected and remote lung parenchyma in a patient with acute COVID-19 pneumonia [5]. The aim of the present study was to expand that preliminary finding, by applying a systematic assessment of native T1 and T2 lung tissue mapping in a cohort of patients with COVID-19 pneumonia and comparing the results to those obtained in a sample of healthy controls.

## 2. Materials and Methods

A detailed description of patients’ enrollment protocol at our institution has been previously described [6]. In the present study, we included *n* = 11 COVID-19 patients in which cardiac MRI imaging was performed during in-hospital stay, which was directed by a guidelines-based clinical indication. An age- and sex-matched group of individuals (*n* = 11) was randomly selected among non-COVID-19 patients who underwent cardiac magnetic resonance (CMR) at our institution in the past year. Imaging protocol included gradient-echo scout sequences (TRUFI) acquired in the coronal, sagittal and axial planes covering all the chest, cine sequences (4 chamber, 2 chamber and 3 chamber long axis as well as short axis views), and native T1 and T2 mapping, which were all acquired using the same positions (three short-axis planes at basal, mid and apical and a 4-chamber view). Native T1 mapping was performed by using a modified Look-Locker inversion recovery (MOLLI) sequence with parameters set as follows: TE/TR/flip angle: 1.8/306.20/35°, voxel size 1.6 × 1.6 × 8 mm, base resolution 256, MOLLI scheme 5(3)3 with 8 raw images obtained according to different inversion times. T2 mapping was performed by using a T2-prepared steady-state free precession pulse sequence with the following parameters: three T2W images with different T2 preparation times (0 ms, 25 ms, 55 ms), repetition time 3 × RR, acquisition time 7 × RR, image matrix 76 × 256, single shot acquisition, flip angle 70°, parallel acquisition (acceleration) GRAPPA.

**Figure 1:**
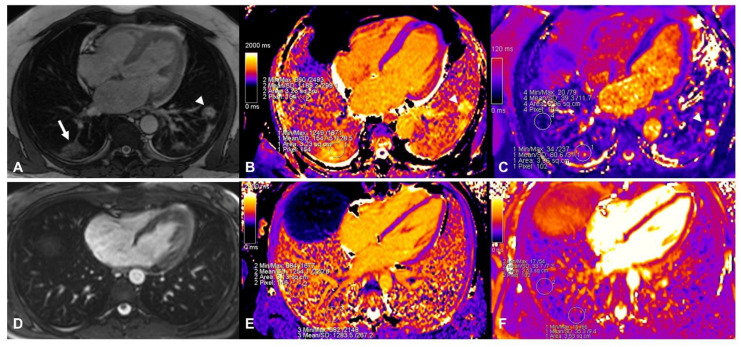
Assessment of native T1 and T2 mapping in a patient with COVID-19 (top panels). The affected area was identified in TRUFI sequence (white arrow in (**A**), another involved area was found in the left lung indicated by the arrowhead) and ROIs were placed in the same area in native T1 (panel (**B**), affected parenchyma T1: 1547 ms; remote parenchyma T1: 1189 ms) and T2 (panel (**C**), affected parenchyma T2: 81 ms; remote parenchyma T2: 39 ms) mapping images. In the bottom panels the assessment of lung tissue mapping in the corresponding control is depicted. TRUFI imaging revealed no abnormal areas within the lung parenchyma (panel (**D**)), similar ROIs as in the patient with COVID-19 depicted in the top panels were placed in native T1 (panel (**E**), 1254 ms and 1284 ms) and T2 (panel (**F**), 34 ms and 35 ms) images.

**Figure 2:**
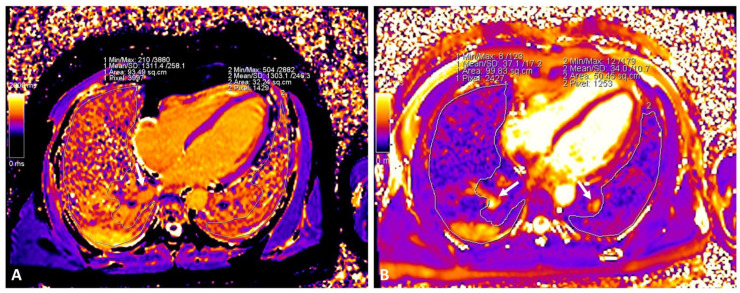
Assessment of native global native T1 and T2 lung mapping in a patient with COVID-19 ((**A**): native T1; (**B**): T2). Large vessels (arrows) were excluded from the ROI.

Lung native T1 and T2 mapping assessments were performed by drawing a circular region of interest (ROI) with a diameter of 2 cm in the parenchyma visualized from the cardiac 4 chamber long axis-oriented slice. Vessels and areas of pleural effusion were carefully excluded. As previously reported [5], the remote and affected areas in COVID-19 were identified by mapping raw images or TRUFI sequence (Figure 1A), and ROIs were drawn accordingly (Figure 1B,C). In each corresponding control, a similar approach was applied with ROIs drawn in corresponding areas (Figure 1D–F). Additionally, global lung parenchyma T1 and T2 values were calculated by drawing large ROIs within both lungs, avoiding main vessels and areas of pleural effusion (Figure 2A,B). As previously described [7], myocardial native T1 and T2 mapping were assessed by drawing an ROI within the mid-ventricular LV septum. Blinded re-reading of the mapping images was performed by a second expert operator to test for the reproducibility of a native T1 and T2 assessment of the lung parenchyma with a calculation of an interclass correlation coefficient. Receiver operator characteristics (ROC) curves were built to assess the ability of different lung tissue mapping approaches in differentiating COVID-19 patients against the controls. Statistical analysis was performed with SPSS version 25.0. Chi-square, Fisher exact test, Mann–Whitney U test or Kruskal–Wallis test, as appropriate, were used to compare groups. A two-tailed *p* < 0.05 was considered statistically significant.

## 3. Results

**Figure 3:**
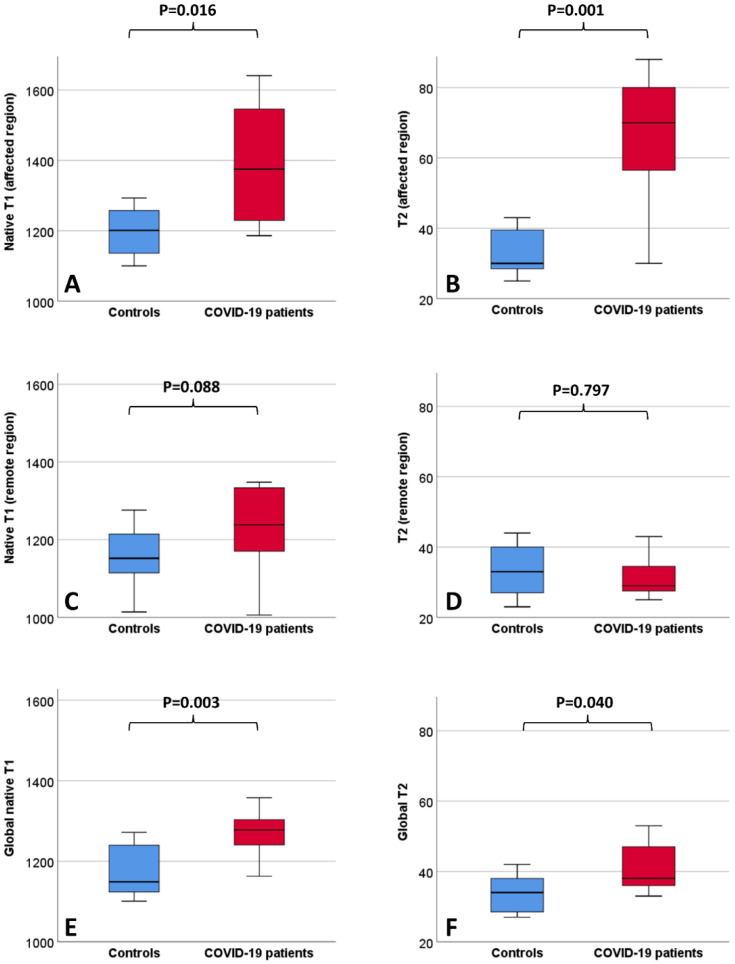
Boxplots depicting lung MRI tissue mapping findings in the healthy controls and COVID-19 patients. Values (ms) refer to native T1 (**A**) and T2 (**B**) of the affected regions, native T1 (**C**) and T2 (**D**) of the remote regions and to native T1 (**E**) and T2 (**F**) of the whole lung parenchyma.

**Figure 4:**
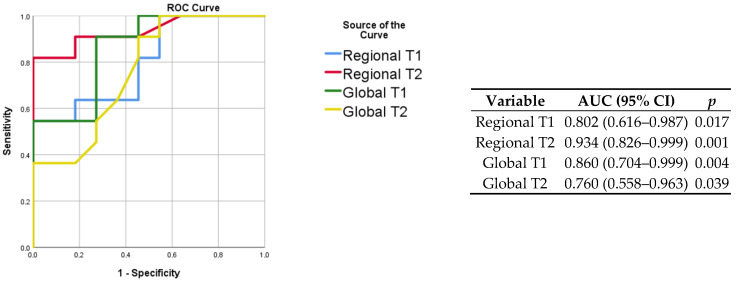
ROC curves for the prediction of COVID-19 status (**left**) with corresponding discriminatory value (**right**). The highest AUC was obtained when assessing the regional T2 of affected lung regions.

The baseline clinical characteristics of the enrolled patients and MRI findings are summarized in Table 1. No patients had a previous history of cardiovascular disease (including known coronary artery disease, heart failure, cardiomyopathy, atrial fibrillation). As compared to the controls, patients with COVID-19 had similar cardiac function and higher septal myocardial native T1 (1028 ms vs. 985, *p* = 0.05). Lung tissue mapping analysis revealed significantly higher native T1 and T2 values in the COVID-19 patients as compared with the controls, when the ROI was placed to include the area visually affected by pneumonia (1375 ms vs. 1201 ms, *p* = 0.016 and 70 ms vs. 30 ms, *p* < 0.001, respectively); conversely, non-significant differences were observed between the remote lung areas of the patients and the controls (1238 ms vs. 1152 ms, *p* = 0.088 and 29 ms vs. 33 ms, *p* = 0.797, respectively) (Figure 3). When a larger ROI was drawn, comprising the whole lung parenchyma within the image and irrespective of the location of the affected and remote areas, the COVID-19 patients still had higher native T1 (1278 ms vs. 1149 ms, *p* = 0.003) and T2 values (38 ms vs. 34 ms, *p* = 0.04) (Figure 3). The ROC analysis results are summarized within Figure 4. Both the regional and global T1 and T2 mapping assessment retained significant diagnostic accuracy, albeit with variable discriminatory values. We found a variable agreement between the readers in the evaluation of T1 and T2 lung tissue mapping in the COVID-19 patients. Better results for the global rather than regional assessments were found. The interclass correlation coefficients were as follows: regional T1 (affected) 0.875 (95% confidence interval [CI] 0.604–0.965, *p* < 0.001); regional T1 (remote) 0.198 (95% CI −0.427–0.694, *p* = 0.269); regional T2 (affected) 0.484 (95% CI −0.127–0.829, *p* = 0.055); regional T2 (remote) 0.552 (95% CI −0.035–0.856, *p* = 0.031); global T1 0.963 (95% CI 0.870–0.990, *p* < 0.001); global T2 0.984 (95% CI 0.940–0.996, *p* < 0.001).

## 4. Discussion

To the best of our knowledge, this is the first report describing lung MRI T1 and T2 mapping findings in hospitalized patients with COVID-19 pneumonia and healthy controls. Native T1 and T2 values of the lung parenchyma were higher in the patients than in the controls, either when using a regional or global measurement approach. The T2 value of the affected regions retained the best diagnostic accuracy to differentiate the patients against the controls, whereas a higher reproducibility was obtained when assessing the global lung native T1 and T2 values. Myocardial native T1 was higher in the COVID-19 patients than in the controls.

MRI mapping techniques are increasingly used to characterize myocardial changes in a variety of clinical conditions [7,8,9], including COVID-19 [10], and this is consistent with our observation of the increased myocardial native T1 values detected in the patient group. On the other hand, few studies are currently available regarding tissue characterization when using a lung MRI, of which none of them include COVID-19 patients. Gargani et al. [3] reported data from a cohort of patients with systemic sclerosis, observing an increased signal by using T2-weighted STIR sequences, consistent with the presence of edema and, thus, inflammation. Previous studies investigating lung T1 mapping in pediatric patients with cystic fibrosis found reduced values as compared to controls, which is possibly explained by the presence of reduced blood flow due to fibrosis or vasoconstriction in the affected areas [4]. Together, these studies demonstrate the ability of MRI in detecting abnormalities within lung tissues in diverse clinical conditions, mainly due to the different fluid distribution, either blood or edema. In our study, we observed the concomitant increase of native T1 and T2 values in the affected lung areas of the patients with COVID-19 pneumonia. This finding should be read as secondary to the presence of extensive edema, since the presence of water influences both the rise of the specific marker T2 as well as the native T1 [11]. The presence of edema is consistent with acute pneumonia and ongoing lung inflammation, which is also one of the therapeutic targets during the acute phase of COVID-19 [12]. Results from our study suggest that native T1 and T2 mapping could be potentially used not only for the identification but also parametric quantification of the ongoing inflammation of lung parenchyma. To date, scarce data are available in the literature regarding the optimal approach for the tissue mapping evaluation of the lungs. If, on the one hand, the regional assessment could be more informative regarding local pathologic processes within the affected areas in the course of pneumonia, this is achieved at the expense of a lower reproducibility of the measure, as indicated by the results of the blinded re-reading of the images in our sample. Translating the evidence obtained in the field of cardiovascular diseases by using cardiac MRI, it might be assumed that better results with tissue mapping techniques should be obtained when applying them to diffuse rather than regional diseases, when the entire architecture of the organ is affected [13]. Notwithstanding this, the ROC curves from our study suggested a potential diagnostic role for the global lung mapping assessment too, that could be explained by the relatively diffuse nature of COVID-19 pneumonia as compared to other subtypes, such as those of bacterial origin. One limitation of this report was the small sample size that prevented a comprehensive description of any existing association between native T1 and T2 with pneumonia severity, systemic inflammation and outcome. Furthermore, where active inflammation is likely the cause of the detected MRI abnormalities, further studies are needed to assess whether lung MRI T1 and T2 mapping could be effectively used to image the long-term consequences of COVID-19 pneumonia after recovery, including fibrosis [14]. T1 and T2 mapping techniques can provide an accurate tissue characterization; however, they are limited by the need for a standardized setup for the imaging and definition of normal values [15], with variable results obtained when using different field strengths, machine vendors and/or post-processing software, which may limit the generalizability and immediate transferability of the absolute quantitative values reported here.

## 5. Conclusions

In our cohort of patients with COVID-19, T1 and T2 mapping lung MRI identified pneumonia-related abnormalities, likely representing edema and ongoing inflammation of the lungs. Measuring T2 mapping within the affected areas seems to retain the best diagnostic accuracy, though the global assessment of the whole lungs provided higher reproducibility. Myocardial native T1 was higher in the COVID-19 patients than in the controls, indicating the concomitant cardiac involvement.

## Figures and Tables

**Table 1:** Characteristics of the study population. Chronic obstructive pulmonary disease (COPD), cardiovascular disease (CVD), hemoglobin (Hb), C-reactive protein (CRP), fibrinogen equivalent unit (FEU), magnetic resonance imaging (MRI), left ventricular ejection fraction (LVEF). Bold indicates *p* < 0.05. * Including known coronary artery disease, heart failure, cardiomyopathy, atrial fibrillation.

Variable	COVID-19 (*n* = 11)	Controls (*n* = 11)	*p*
Age (year old)	55 (46, 76)	53 (44, 77)	>0.99
Male sex (%)	7 (64)	7 (64)	>0.99
**Coexistent Conditions**			
Hypertension (%)	2 (18)	-	-
Dyslipidemia (%)	2 (18)	-	-
Diabetes (%)	1 (9)	-	-
COPD (%)	1 (9)	-	-
Previous CVD * (%)	0 (0)	-	-
**Laboratory Tests**			
Hb (g/dL)	13.7 (11.8, 14,2)	-	-
Lymphocyte (per µ109/L)	1.2 (0.9, 1.8)	-	-
Creatinine (mg/dL)	0.9 (0.6, 1)	-	-
CRP (mg/dL)	3.6 (0.4, 14)	-	-
D-dimer (ng/mL FEU)	1027 (434, 1679)	-	-
Hs-Troponin (pg/mL)	11 (6, 135)	-	-
**Blood Gas Analysis**			
PaO_2_/FIO_2_	346 (251, 415)	-	-
**MRI findings**			
LVEF	60 (56, 63)	61 (59, 65)	0.332
T1 myocardium (mid-septum)	1028 (972, 1058)	985 (962, 993)	**0.05**
T2 myocardium (mid-septum)	45 (43, 48)	45 (44, 47)	0.949
Lung T1 mapping			
Global	1278 (1238, 1313)	1149 (1107, 1249)	**0.003**
Remote	1238 (1165, 1337)	1152 (1114, 1249)	0.088
Affected	1375 (1220, 1580)	1201 (1130, 1270)	**0.016**
Lung T2 mapping			
Global	38 (36, 49)	34 (28,39)	**0.040**
Remote	29 (27, 35)	33 (27, 41)	0.797
Affected	70 (55, 82)	30 (28, 40)	**0.001**

## Data Availability

Data will be made available upon reasonable request.
